# A Five-Year Analysis of Industry Payments to Sleep Neurologists From 2014 Through 2018

**DOI:** 10.7759/cureus.10597

**Published:** 2020-09-22

**Authors:** Vasuki Dandu, Suman Siddamreddy, Vaishali Thombre, Karthika Durga Veerapaneni, Sisira Yadala, Sen Sheng, Ruchira Mahashabde, Yohei Harada, Nidhi Kapoor, Sanjeeva Onteddu, Krishna Nalleballe

**Affiliations:** 1 Neurology, Baptist Health Medical Center, Little Rock, USA; 2 Internal Medicine, Baptist Health Medical Center, Little Rock, USA; 3 Biostatistics and Epidemiology, University of Arkansas for Medical Sciences, Little Rock, USA; 4 Neurology, University of Arkansas for Medical Sciences, Little Rock, USA; 5 Neurology/Stroke, University of Arkansas for Medical Sciences, Little Rock, USA

**Keywords:** sleep, neurology, payments from industry

## Abstract

Background and objectives

Sleep medicine has been one of the fastest-growing medical fields in recent years. The industry plays a big role in developing new medications and devices for both diagnosis and treatment of sleep-related problems. We analyzed payments made by industry to physicians from 2014 through 2018 based on the Open Payments Program data.

Methods

Centers for Medicare and Medicaid Services Open Payment Program and American Board of Psychiatry and Neurology databases were explored to elicit financial relationships between industry and sleep neurologists.

Results

Payments made by industry to sleep neurologists have been steadily increasing from 2014 through 2018. Approximately 16% to 22% of sleep certified neurologists received payments from industry during the study period. Interestingly, the payments made to the top 10% of the sleep physicians contributed approximately 85% to 96% of the total payments. The top two categories to which the highest payments were made were compensation for services and royalty and/or licensing fees. Silenor® (doxepin), Xyrem® (sodium oxybate), Aptiom® (eslicarbazepine acetate), Belsomra® (suvorexant), and Fycompa® (perampanel) were most of the drugs, which made the highest payments, that got approved by the Food and Drug Administration in the last decade.

Conclusions

It seems that the industry is spending significant amounts of money in educating the physicians and in marketing the newer drugs. This analysis of the data on payments from industry is very useful in identifying any potential conflicts of interest from physicians. Further analyses are needed to study the trends of physician practice behavior and decision making.

## Introduction

Relationships between industry and physicians have been established for many years. Lately, it has been an area of great interest to the medical community and patients. Many studies describe the physician behavior pattern in clinical, research, and educational domains based on their association with industry payments.

One study reported that industry spent $1.2 billion (United States dollar, USD) to Continuing Medical Education (CME) activities that constituted 50% of all CME funds across the country in just 2007 alone [1]. Physicians who received more payments from the industry have been shown to prescribe more branded medications. Additionally, CME-sponsored events by industry have been shown to affect the prescribing patterns among physicians by preferentially highlighting their products [2]. However, the undue influence of industry payments on physician decision making is still not very clear and needs further research and analysis.

Informing customers with mandatory disclosure of conflicts of interest is a strategy practiced not only in the medical field but also in finance and accounting professions. On the other hand, there is a need for physician-researcher collaboration with industry for developing novel treatment options. Empirical evidence relevant to conflicts of interest and industry payments is lacking. As most of the studies are observative rather than interventional, these issues cannot be investigated using randomized control trials.

The Open Payment Program (OPP) was initiated in 2007 under the Physicians Payment Sunshine Act (PPSA) and enacted in 2010 under the Affordable Care Act. Under the OPP, the Centers for Medicare and Medicaid Services (CMS) publicly reports payments made by drug and device manufacturers and group purchasing organizations to physicians and teaching hospitals.

Sleep medicine is one of the fastest advancing technological fields in medicine. The growth of mobile technology (e.g., consumer applications, telemedicine, and wearable devices) is helping physicians to better diagnose and treat sleep problems.

To the best of our knowledge, financial relationships between industry and sleep neurologists have not been reported previously. We analyzed industry payment amounts, percentages, and categories and identified trends among various drugs, regions in the US, and types of compensation from 2014 through 2018.

## Materials and methods

Data sources

A retrospective analysis of the CMS OPP database (https://openpaymentsdata.cms.gov/query-builder) was accessed from the physician specialty category “Allopathic & Osteopathic Physicians, Psychiatry & Neurology, Sleep Medicine”, and data were collected on industry payments to sleep neurologists from January 1, 2014 through December 31, 2018. Another publicly available database, the American Board of Psychiatry and Neurology (https://www.abpn.com/wp-content/uploads/2016/08/ABPN-Certifications-by-Year-Subspecialties.pdf ), was also accessed to gather data on sleep neurologists. There is no risk of double-counting because both databases are entirely independent.

Primarily, we divided the industry payments into three broad categories: general, research, and ownership. Research and ownership payments were excluded from our study because those payments were either too small to categorize or not present.

The general payment category was further subdivided into 10 subcategories: compensation for service (e.g., speaking at a noncontinuity medical education program), consulting fees, education, entertainment, food and beverage, gift, grant, honoraria, royalty and/or licensing fees, and travel and lodging. Each payment was attached to a particular physician associated with a unique ID (Identification), state of residence, and drug (with the sponsored company).

For easier analysis, we divided the US into five geographical regions: Midwest, South, Northeast, West, and Puerto Rico. The top 10 drugs associated with each year's highest payments were identified and included in our analysis.

Data analysis

The total number of sleep physicians was obtained from the American Board of Psychiatry and Neurology database. Using that as a denominator, the percentage of total sleep physicians who obtained industry payments was calculated [3]. For general payments, mean, median, standard deviation, interquartile range, and Gini index were calculated. The Gini index ranges from 0, indicating every physician received an equal number of payments, to 1, indicating that one physician received all the payments and none of the others received any payment.

We also identified the top 10% of sleep neurologists as those who received the highest amount of industry payments each year along with the top 10 physicians who received the highest payments each year; their percentage of total payments was calculated. Payments to subcategories of general payment were calculated for each year and included in the analysis. Trends in payments based on the geographical region were also analyzed.

## Results

Distribution of payments

The sum of all payments from industry to sleep neurologists from 2014 through 2018 was calculated as $4,665,100. Out of which, the total for general payments equaled $4,580,681. The general payments showed a steady trend throughout the study period, contributing to approximately 97.3% to 98.5% of the total for all payments (Table 1).

**Table 1:** Type of payments from 2014 through 2018

Year	General Payment (%)	Research Payment (%)	Ownership Payment (%)	Total
2014	$457,270 (97.3%)	$12,874 (2.7%)	0	$470,144
2015	$651,111 (98.3%)	$11,107 (1.7%)	0	$662,218
2016	$917,616 (98.2%)	$16,575 (1.8%)	0	$934,191
2017	$1,281,495 (98.9%)	$13,881 (1.1%)	0	$1,295,376
2018	$1,273,189 (98.5%)	$19,982 (1.5%)	0	$1,293,171
Total	$4,580,681 (98.4%)	$74,419 (1.6%)	0	$4,655,100

The percentage of physicians who received payments varied from 16% to 22% of the total certified sleep neurologists. Initially, there was an increase from 18% in 2014 to 22% in 2015. In 2016, the percentage decreased to 16% and remained stable at 16% after that. However, the total value of the payments increased significantly from $457,270 in 2014 to $1,273,189 in 2018.

During the study period, the median payment remained fairly stable ($117-$140), but the mean payments varied much more ($1,844-$5,340). Also, the maximum payment made each year showed a large variation from $274,913 in 2014 to $468,571 in 2018 (Table 2).

**Table 2:** Characteristics of general payments from 2014 through 2018 * Percentage is calculated as the number of physicians received payment divided by the number of board-certificated physicians in sleep. † Gini index ranges from 0 (every physician received an equal number of payments) to 1 (one physician received all the payments and none of the others received any payment) ‡ Top 10% physicians are defined as those who received the highest amount of industry payments each year among all sleep physicians who received payments. § Top 10 physicians are defined as those top 10 physicians who received the highest payments each year.

Payment Descriptive	2014	2015	2016	2017	2018
Number of physicians received payment	248	312	240	240	247
Number of the board-certified physicians in sleep	1,398	1,398	1,502	1,502	1,580
Percentage of recipients^*^	18%	22%	16%	16%	16%
Total value of payment	$457,270	$651,111	$917,616	$1,281,495	$1,273,189
Mean payment	$1,844	$2,087	$3,823	$5,340	$5,155
Standard deviation (SD)	$17,558	$7,657	$23,932	$30,687	$33,295
Median payment	$117	$140	$112	$100	$122
Interquartile range (IQR)	$355	$487	$330	$271	$292
Gini index^†^	0.93	0.88	0.94	0.95	0.95
Total payment from the top 10% physicians^‡^	$414,922	$556,548	$859,861	$1,225,516	$1,217,299
Payment percentage of the top 10% physicians	91%	85%	94%	96%	96%
Maximum payment	$274,913	$80,926	$328,338	$399,659	$468,571
Total payment of Top 10 physicians^§^	$375,744	$382,877	$770,043	$1,106,121	$1,082,156
Payment percentage of the top 10 physicians	82%	59%	84%	86%	85%

Categories of payments

The highest-paid category was compensation for service, which amounted to 41.8% of the total payments for the study period. It consists of mainly the speaker fees at a noncontinuity medical education program (Table 3).

**Table 3:** Categories of general payment

Category	2014	2015	2016	2017	2018	Total
Compensation for service	$26,725	$372,362	$391,659	$622,907	$500,452	$1,914,105
Consulting fee	$65,413	$35,957	$22,670	$19,555	$53,570	$197,164
Education	$7,541	$8,050	$1,167	$5,458	$1,055	$23,271
Entertainment	$1,090	$351	$161	$100	$0	$1,703
Food and beverage	$52,694	$104,427	$69,965	$78,689	$79,513	$385,288
Gift	$2,025	$1,010	$265	$81	$1,040	$4,421
Grant	$0	$0	$0	$0	$55,000	$55,000
Honoraria	$2,535	$52,304	$23,650	$23,838	$11,188	$113,514
Royalty or license	$274,913	$420	$329,630	$413,990	$471,317	$1,490,269
Travel and lodging	$24,335	$76,230	$78,448	$116,878	$100,052	$395,943
Total	$457,270	$651,111	$917,615	$1,281,495	$1,273,186	$4,580,678

The second most paid category was royalty and/or licensing fees (32.5%). Of total payments, travel and lodging amounted to 8.6%. Even though food and beverage were the most frequently made payment (17,307 transactions in five years), it only constituted 8.4% of total payments. 

Top 10 payment receivers and top 10% payment receivers

The amount paid to the physicians who were in the top 10% increased from $414,922 (91% of the total payment) in 2014 to $1,217,299 (96% of the total payment) in 2018. Of note, the payments made to physicians who stood among the top 10 comprised approximately 82% to 86% of total payments from the industry for all years except 2015. Because of this, the Gini index was close to 0.93 to 0.95 for the most years, suggesting inequality among physicians who received payments (Table 2).

Drugs 

During the study period, the top five drugs that made the most payments were Silenor® (doxepin), Xyrem® (sodium oxybate), Aptiom® (eslicarbazepine acetate), Belsomra® (suvorexant), and Fycompa® (perampanel). We did not notice any device companies that made significant payments to the physicians.

Geographical variation

The industry spent more money on sleep neurologists in the South region (39.3%), followed by Northeast (35%), West (12.8%), Midwest (12.6%), and Puerto Rico (0.03%). This trend of the highest payments being in the South is also seen in Vascular Neurology payment studies [4].

## Discussion

Sleep medicine is comparatively a newer field in the vast array of medicine. Several medical specialties, such as internal medicine, neurology, family medicine, radiology, and otolaryngology, have a pathway to obtain subspecialty certification in sleep medicine. Approximately 25 million US adults (13.08% of the total US population) have obstructive sleep apnea, and 30% suffer from insomnia. The numbers show that there is a need for research and development in the field of sleep medicine. Many new medications were approved for sleep-related disorders in recent years. Industry plays a major role in marketing and physician education of these newer drugs. Our study showed that the top five drugs that made the highest payments were all approved by the Food and Drug Administration (FDA) in the last decade, except for Xyrem (sodium oxybate). However, the FDA approved Xyrem (sodium oxybate) for narcolepsy in the pediatric population in October 2018, which probably caused the company to spend more money on marketing in recent years.

Our study showed that over the years, the industry spent more money to reach more physicians. This was reflected in the trend of compensation for service and royalty and/or licensing fees being the top categories on which the industry spent money.

In our analysis, the top 10 physicians received close to 85% of total payments, which shows a gross inequality among the physicians who received payments from the industry. The same trend has been noted in other studies analyzing industry payments to cardiologists and neurologists [5-10]. The trend of payments in the top 10 physicians was not much different from the total cohort; compensation for services (41.3%) and royalty and/or licensing fees (31.6%) were the most paid categories. 

Other specialties like orthopedic surgeons and neurosurgeons who depend on devices have been shown to receive high payments from the device industry [11-14]. Interestingly, in our analysis, we did not notice many device manufacturers that made payments to physicians despite sleep medicine being heavily dependent on devices both for diagnosis and treatment of sleep disorders. Our study did not explore the reason behind that as it was not pertinent to our study.

Limitations

Sleep medicine is a new specialty, and because several medical specialties have a path to obtain a certification in that field, this might cause an error in reporting that could alter the analysis of industry payments to sleep neurologists.

Our study also noticed that there are several medications such as Aptiom (eslicarbazepine acetate) and Fycompa (perampanel) that have primary indications for partial seizures and have made high payments to sleep neurologists. Our analysis showed that several epilepsy-certified neurologists are also certified in sleep medicine, making it difficult to filter those transactions based on the subspecialty. 

The OPP is a self-reporting database, and its accuracy is not completely known. Even though the quality of the OPP data has improved over time, there could still be inaccuracies [15]. Additionally, there is a time in which a physician or a hospital can dispute a payment record. Because of the rarity of disputes, the validity of the data cannot be considered completely accurate.

Under the PPSA, the annual penalty is capped at $150,000 for an unintentional failure to report and $1 million for an intentional failure to report [16]. Profitable companies might not be deterred by these penalties and might not completely report, making the data incomplete. We feel that there is a great need for additional research and analysis of industry payments on the physician's influence in delivering care for their patients.

## Conclusions

Even though the payments from industry to sleep neurologists have more than doubled from 2014 through 2018, only the top 10% of sleep neurologists are receiving a major part of the total share. The South region leads to the number of payments received to sleep neurologists by industry. The top five drugs that made payments were all approved by the FDA in the last 10 years or so. It seems to reflect that industry is trying to spend more money on increasing awareness of newer drugs.
